# Perception of stress level, trunk appearance, body function and mental health in females with adolescent idiopathic scoliosis treated conservatively: a longitudinal analysis

**DOI:** 10.1007/s11136-012-0316-2

**Published:** 2012-11-28

**Authors:** Ewa Misterska, Maciej Glowacki, Joanna Latuszewska, Katarzyna Adamczyk

**Affiliations:** 1 Department of Pediatric Orthopaedics and Traumatology, Poznan University of Medical Sciences, ul. 28 Czerwca 1956 135/147, 61-545 Poznan, Poland; 2 Department of Motor System Rehabilitation, Poznan University of Physical Education, ul. Królowej Jadwigi 27/39, 61-871 Poznan, Poland; 3 Department of Human Development Psychology and Family Studies, Adam Mickiewicz University, ul. Szamarzewskiego 89, 60-568 Poznan, Poland

**Keywords:** Longitudinal analysis, Spinal deformity, Body image, Mental health, Stress, SRS-22, TAPS, BSSQ

## Abstract

**Purpose:**

In the presented study, we aimed to assess changes over time in the perception of trunk deformity, body function, stress level and mental health in females with adolescent idiopathic scoliosis (AIS) who were treated conservatively with a Cheneau brace, taking the Trunk Appearance Perception Scale (TAPS), Scoliosis Research Society-22 (SRS-22) and Bad Sobberheim Stress Questionnaires (BSSQ) criteria of evaluation into consideration.

**Methods:**

The study design was comprised of three questionnaire assessments, with the second and the third evaluation taking place 6 and 12 months after the beginning of the study, respectively. Thirty-six females treated conservatively were asked to fill in the TAPS, SRS-22 and BSSQ forms.

**Results:**

In regards to TAPS, the results differed between the 1st and the 2nd assessment in Figure 2 only (*p* = 0.013). The difference between the 1st and the 3rd evaluation concerned Figure 3 and the total score (*p* = 0.011 and *p* = 0.005, respectively). The SRS-22 and BSSQ results of study participants did not differ significantly between the 1st and the 2nd, between the 2nd and the 3rd and between the 1st and the 3rd evaluations.

**Conclusions:**

The study indicated that the assessment of girls with AIS concerning body function and mental health did not deteriorate in the course of orthosis treatment. Furthermore, they showed improvement in perceptions particularly in regards to trunk shape. We pointed out that the negative perceptions of mental health, self-image and low level of activity held by females with AIS coexisted with severe emotional distress. Moreover, factors that improved functioning or subjective physical appearance ratings in particular, such as level of activity, were indicated.

## Introduction

Emotional burden and anxiety in particular are the most frequent issues in social situations reported by people with disfiguring conditions [1]. Moreover, research has demonstrated that body disfigurement, which can be seen in adolescent idiopathic scoliosis (AIS), can have a consistent negative effect on the development of an individual’s body image. This, in turn, can additionally result in decreased self-esteem and social confidence along with increased anxiety, depression and stress [2–6], particularly in female adolescents undergoing brace treatment [7–10].

Other studies suggested that bracing treatment had a psychological impact, causing low self-esteem and a more negative self-image, but no psychopathological changes in the long term [11, 12]. Psychological stress is a cause of special concern for professionals, as it can correlate with a lack of compliance to brace treatment from females with AIS [6, 13]. The Reichel and Schanz’s report of psychological observations of females revealed that in the course of brace treatment, one of the main concerns they expressed was making a visually negative impression on others due to the orthosis [7].

Until now, most of the analyzed research consisted of long-term follow-up studies assessing the effectiveness of bracing in terms of scoliosis progression. Considering the sex distribution of AIS, it must be emphasized that scoliosis affects more adolescent females than males so that the sex ratio from age 10 onward is 6:1 [14]. Furthermore, the results of Payne et al. [13] and a review of the studies carried out by Eliason and Richman [15] indicated that male–female differences exist in reactions to body deformation due to scoliosis and treatment methods. This might be due to the fact that higher Cobb angle values related to scoliosis occur more frequently in females with AIS [13].

The role of appearance-specific cognitions in influencing levels of distress in females with disfiguring conditions must be emphasized [16], since spinal disfigurement is one of their greatest concerns and a primary objective of treatment. However, while disfigurement usually appears to have a negative impact great variations do exist among individuals [2, 3].

In this study, we aimed to assess changes over time in the perception of trunk deformity, body function, stress level and mental health in females with AIS who were treated conservatively with a Cheneau brace, taking the Trunk Appearance Perception Scale (TAPS), Scoliosis Research Society-22 (SRS-22) and Bad Sobberheim Stress Questionnaires (BSSQ) [4–6, 17–22] criteria of evaluation into consideration.

## Materials and methods

The study design was prospective and was comprised of three clinical and questionnaire assessments. The radiological evaluation was performed during the first and the third assessments only. The second and the third evaluations took place 6 and 12 months, respectively, after the beginning of the study.

The TAPS, SRS-22, BSSQ-Brace and BSSQ-Deformity [4, 22] were administered during a routine patient visit. The investigator was available throughout the visit should participants require explanation or clarification. All the female participants received detailed information on the aim of the study and were assured of anonymity, following which they gave their informed consent. The study was approved by the Bioethics Committee of Poznan University of Medical Sciences.

### Sample size and selection criteria

The final study group was comprised of 36 consecutively selected females with AIS treated conservatively with a Cheneau brace and recruited from one center (Department of Pediatric Orthopaedics and Traumatology, Poznan University of Medical Sciences, Poland). The inclusion criteria were as follows: a minimum daily Cheneau bracing time of 12 h per day (information based on the clinical examination), a Cobb angle of 20°–40°, 10–17 years of age and a Risser sign from 0 to 2 at the beginning of brace treatment. The participant selection period lasted for 20 months, from July 2010 to February 2012. We excluded girls in whom other diseases leading to trunk deformity or serious medical conditions were diagnosed.

Forty-five females with AIS were eligible for the study, and two participants dropped out after 6 months. They did not give reasons for nonparticipation. One male patient came forward but was excluded from the study, for reasons of insufficient male group sample size. After 12 months, a further 6 females dropped out of the study, one female was unavailable for follow-up evaluation because she was experiencing cardiac problems, whereas the remaining 5 did not provide an explanation.

Considering duration of brace wearing in months, the first evaluation of female study participants took place at a mean of 17.9 months SD 17.6, the second at 24.5 months SD 17.5 and then 30.1 months SD 17.6 at the final assessment after beginning Cheneau brace treatment. In addition, after grouping the subjects by duration of brace wearing in months, we indicated 19 girls (52.8 %) wore the brace for 0–12 months, 6 (16.7 %) wore the brace for 13–24 months and the remaining 11 girls (30.5 %) wore the brace for >24 months. Considering daily bracing time, on average, the girls wore the brace for 15.9 h SD 2.9 (first evaluation), 15.6 h SD 2.6 (second) and 15.2 h SD 2.2 (third and final evaluation) per day.

### Measurement instruments

#### The Trunk Appearance Perception Scale

The TAPS is a scale that evaluates the degree of trunk deformity and includes three sets of figures that depict the trunk from 3 viewpoints: a back view (Figure 1), a view of the patient bending forward seen from the front (Adam’s test), (Figure 2) and a frontal view (Figure 3). This last view has two sets of drawings, one for males and one for females. Each drawing is scored from 1 (greatest deformity) to 5 (smallest deformity) and a mean score is obtained by adding the scores for the 3 drawings and dividing the total by 3 [20]. The internal consistency of the TAPS (Cronbach’s alpha coefficient 0.89) and test–retest reliability (ICC 0.92) are excellent. The convergent validity of the TAPS, measured by the correlation between the TAPS score and SRS-22 score, revealed all Spearman’s correlation coefficients were statistically significant (*p* < 0.01), although the SRS-22 scores showing the highest correlation with the TAPS were the self-perceived body-image scale (range, 0.43–0.54) and the total score (range, 0.45–0.52) [20]. Since our study represents the first use of the Trunk Appearance Perception Scale in the Polish population, we analyzed the psychometric properties of TAPS in the studied group of brace-treated scoliosis females.

#### The SRS-22 Patient Questionnaire

This is a disease specific questionnaire which is accepted as a simple and practical means of obtaining a patient’s perception of scoliosis. The SRS-22 contains 22 questions that cover 5 domains: function/activity, 5 items; pain, 5 items; self-perceived body image, 5 items; mental health, 5 items and satisfaction with treatment, 2 items. The scores for each answer range from 1 (worst) to 5 points (best) and in each domain the recipient can score from 5 to 25 points, except for the *satisfaction from treatment* subscale where they can score from 2 to 10 points [17–19, 22]. However, each domain as well as the total score is often expressed as the average of all item responses and, therefore, the range is from 0 to 5 points, with a higher score indicating a better outcome [23]. The SRS-22 questionnaire is reliable with an internal consistency (Cronbach alpha) and reproducibility (intraclass correlation coefficient) comparable with SF-36. Internal consistency of SRS-22 ranged from 0.92 to 0.75, whereas reproducibility ranged from 0.96 to 0.85. Concurrent validity, determined by Pearson correlation coefficients between SRS-22 and SF-36 domains, was 0.70 or greater (*p* < 0.0001) [17].

#### *The BSSQ*-*Deformity* and the *BSSQ*-*Brace*

These self-administered scales measure the stress produced by the deformity and by brace wearing in adolescents with idiopathic scoliosis and both consist of eight questions. In particular, BSSQ-Deformity relates to the effect of spine deformity on patient social interaction, mood and, as a result, the level of experienced stress, whereas BSSQ-Brace focuses on the psychological burden connected with the necessity for conservative treatment and assesses the extent to which wearing a brace affects mood, distorts social interaction and consequently leads to an increase in stress levels [4–6]. Possible answers on the Bad Sobberheim Stress Questionnaires are marked on a four-point scale: from 0 to 3; the total score ranges from 0 to 24. The following subdivision of the total score is proposed: 0–8 (strong stress), 9–16 (moderate stress), 17–24 (little stress) [4–6]. The BSSQ-Deformity was shown to have a good recurrence (*r* = 0.95) and a sufficient criterion validity (*r* = 0.78), however, the Cronbach alpha was not obtained [6] and this might have been due to the small number of items [5]. The intraclass correlation coefficient between the values of the two measurements of the BSSQ-Brace was 0.88, which shows this questionnaire has sufficient reliability. Considering internal consistency, the Cronbach alpha was 0.97 which means the internal consistency of BSSQ-Brace is high [5].

### Statistics

Descriptive statistics (mean, 95 % confidence intervals, range and standard deviations) were utilized to describe the distribution of the results with respect to statistical quantitative features. With respect to qualitative features, we assigned percentages to the number of units belonging to the described categories of a given feature.

The psychometric properties of the TAPS were determined by the distribution of scores and determination of the floor effect (% of patients with the minimum score) and ceiling effect (% of patients with the maximum score). Internal consistency was examined using Cronbach’s alpha coefficient. Cronbach’s alpha coefficient values were accepted as follows: excellent ≥0.80, adequate 0.70–0.79 and poor <0.70 [24]. Discriminant validity was assessed by calculating the correlation between the total TAPS score and the Cobb angle. In addition, the responsiveness of the TAPS, SRS-22 and BSSQ were determined by means of effect sizes, which were calculated for each measure by dividing the mean absolute change score by the standard deviation of the baseline score. The interpretation of the magnitude of the effect size was based on Cohen’s rule-of-thumb, in which an effect size of 0.2–0.5 was considered as small, 0.5–0.8 as moderate and over 0.8 or greater represented a large effect [25]. Lastly, the minimum clinically important difference (MCID) of the SRS-22 was established by choosing the standard error measurement (SEM), a distribution-based method. An observed change score that exceeded the standard measurement error was considered to reflect an important change. Patients with a change score that did not exceed the measurement error were considered to be clinically stable. In addition, patients were considered to have minimally improved or minimally worse SRS-22 results if their score increased or decreased by one-SEM, respectively [26].

To compare differences between the three time points for the TAPS, SRS-22 and BSSQ scores, a Friedman two-way ANOVA was utilized. Significant omnibus tests were followed-up using multiple Wilcoxon signed-rank tests with a Bonferroni adjustment.

Spearman’s rank-order correlation coefficients were used to evaluate correlations between TAPS, SRS-22, BSSQ results and detailed clinical and radiological characteristics of study participants, such as body mass index, age, Cobb angle, angle of trunk rotation, apical translation and daily and monthly duration of brace wearing, as well as between TAPS, SRS-22 and BSSQ scores. Correlations were defined as strong >0.60, moderate 0.30–0.60 and weak <0.30, respectively [27].

Finally, a logistic regression was utilized to evaluate the influence of the socio-demographic, brace-related and radiological data, on the probability of achieving a “good result” in the TAPS, SRS-22 and BSSQ. Based on the lower and upper quartile distribution of the results, the TAPS total scores were split into two categories: “good result” (from 12 to 15 points) and “poor result” (below 12 points). Also based on the lower and upper quartile distribution of the results, the SRS-22 total scores were split into two categories: “good result” (from 88 to 110 points) and “poor result” (below 88 points). BSSQ-Deformity and BSSQ-Brace general results were likewise split into two categories: “poor results” (from 0 to 16 points) and “good results” (from 17 to 24 points) based on the following subdivision of the total scores: 0–8 (strong stress), 9–16 (moderate stress), 17–24 (little stress). We adopted *p* = 0.05 as the border level of statistical significance; test results with a *p* value exceeding this level were treated as insignificant. Statistical calculations were performed by means of Statistica software.

## Results

### Descriptive statistics

For detailed clinical and radiological characteristics of study participants, see Table 1. Fifty-eight percent had thoracic scoliosis, 33 % thoraco-lumbar scoliosis and the remaining 9 % had lumbar scoliosis. Th8 was the apical vertebra in ten girls; Th9 in four; Th10 in two; Th11 in five and Th12 in five. L1 was the apical vertebra in seven girls; L2 in one and L3 in two girls.

**Table 1:** Clinical and radiological characteristics of adolescent scoliosis females (*n* = 36)

Parameters	Mean (SD)	Range (min–max)	Mean (SD)	Range (min–max)	Mean (SD)	Range (min–max)
	1st evaluation		2nd evaluation		3rd evaluation	
Body mass index	17.4 (1.7)	12.9–21.0	17.8 (1.9)	13.7–22.2	18.1 (1.9)	14.0–22.7
Age (years)	13.4 (1.7)	10–16	14.0 (1.9)	10–17	14.4 (1.7)	11–18
Cobb angle	27.1 (5.1)	20–38	–	–	24.9 (9.1)	5–48
Angle of trunk rotation as measured with Perdriolli’s inclinometer	8.3 (3.1)	3–15	7.8 (3.5)	1–15	6.9 (3.5)	2–15
Apical translation of the central sacral vertical line (CSVL) according to the Harms Study Group (cm)	2.1 (0.9)	0.4–4.0	–	–	2.1 (1.0)	0.2–4.2
Brace (hours/day)	15.9 (2.9)	8–22	15.6 (2.6)	9–22	15.2 (2.2)	9–18
Brace (in months)	17.9 (17.6)	1–69	24.5 (17.5)	5–76	30.1 (17.6)	12–81

Table 2 presents the distribution of the results for the TAPS, SRS-22 and BSSQ in subsequent evaluations. In the 1st completion of the TAPS, patients scored 3.6 SD 0.6, in the 2nd 3.9 SD 0.5 and 4.0 SD 0.4 in the last completion. The mean SRS-22 total score (excluding satisfaction) was 4.2 SD 0.3 in the 1st, 4.2 SD 0.3 in the 2nd and 4.3 SD 0.3 in the last evaluation.

**Table 2:** Trunk Appearance Perception Scale, Scoliosis Research Society-22, Bad Sobberheim Stress Questionnaire-Deformity and Bad Sobberheim Stress Questionnaire-Brace scores distribution-longitudinal analysis

	Mean (SD)	95 % confidence interval (from–to)	Range (min–max)	Mean (SD)	95 % Confidence interval (from–to)	Range (min–max)	Mean (SD)	95 % Confidence interval (from–to)	Range (min–max)
	1st evaluation			2nd evaluation			3rd evaluation		
*Trunk Appearance Perception Scale*									
Figure 1	3.5 (0.7)	3.3–3.7	2–5	3.8 (0.6)	3.6–3.9	2–4	3.8 (0.5)	3.6–4.0	2–5
Figure 2	3.8 (0.8)	3.5–4.1	2–5	4.2 (0.7)	4.0–4.4	2–5	4.1 (0.5)	4.0–4.3	3–5
Figure 3	3.6 (0.5)	3.4–3.8	2–4	3.7 (0.6)	3.5–3.9	2–5	4.0 (0.6)	3.8–4.2	3–5
Total score	3.6 (0.6)	3.4–3.8	2–4.6	3.9 (0.5)	3.7–4.1	2–4.3	4.0 (0.4)	3.8–4.1	3–5
*Scoliosis Research Society-22*									
Function/activity	4.6 (0.4)	4.4–4.7	3.8–5.0	4.6 (0.4)	4.5–4.7	3.6–5.0	4.6 (0.3)	4.5–4.7	3.8–5.0
Pain	4.6 (0.3)	4.5–4.7	3.8–5.0	4.5 (0.5)	4.3–4.7	2.8–5.0	4.6 (0.4)	4.5–4.8	3.2–5.0
Self-image	3.7 (0.5)	3.5–3.8	2.2–4.6	3.7 (0.5)	3.6–3.9	2.6–4.6	3.9 (0.4)	3.7–4.0	3.0–4.8
Mental health	4.1 (0.6)	3.8–4.3	2.4–5.0	4.1 (0.5)	3.9–4.3	2.8–5.0	4.2 (0.5)	4.0–4.4	2.8–5.0
Satisfaction with treatment	4.3 (0.8)	4.1–4.6	1.0–5.0	4.4 (0.6)	4.2–4.6	3.5–3.5	4.4 (0.5)	4.3–4.6	3.5–5.0
Total score	4.2 (0.3)	4.1–4.4	3.5–4.8	4.2 (0.3)	4.1–4.3	3.6–4.7	4.3 (0.3)	4.2–4.4	3.6–4.8
Bad Sobberheim Stress Questionnaire-Deformity	17.7 (5.0)	16.0–19.4	1–24	18.0 (4.1)	16.6–19.4	8–24	18.1 (4.3)	16.6–19.5	4–24
Bad Sobberheim Stress Questionnaire-Brace	13.8 (5.4)	12.0–15.6	0–23	14.1 (5.3)	12.3–15.9	1–22	15.4 (5.5)	13.5–17.3	0–23

Having analyzed the BSSQ scores, during the 1st assessment, patients felt a moderate level of stress regarding conservative treatment: the mean value was 13.8 SD 5.4, however, the stress level related to perceived trunk deformation was low and the mean value was 17.7 SD 5.0. This difference was statistically significant at *p* = 0.001. In the 2nd and 3rd evaluation, patients felt a moderate level of stress connected with conservative treatment (patients scored 14.1 SD 5.3 and 15.4 SD 5.5, respectively) and little stress related to perceived trunk deformation with mean values of 18.0 SD 4.1 and 18.1 SD 4.3, respectively.

As in the 1st evaluation, in the 2nd and in the final evaluation, the stress related to the necessity of wearing a brace was higher than that related to spinal deformation; the difference was significant at *p* = 0.001 and *p* = 0.038, respectively. Table 3 presents the interpretation of scores achieved in the BSSQ-Brace and BSSQ-Deformity questionnaires, in terms of low, medium and high stress level (for details, see Table 3).

**Table 3:** Interpretation of Bad Sobberheim Stress Questionnaire-Deformity and Bad Sobberheim Stress Questionnaire-Brace – distribution of results on 1st, 2nd and 3rd evaluation

Bad Sobberheim Stress Questionnaire Interpretation	Bad Sobberheim Stress Questionnaire-Deformity % (*n*)			Bad Sobberheim Stress Questionnaire-Brace % (*n*)		
	1st evaluation	2nd evaluation	3rd evaluation	1st evaluation	2nd evaluation	3rd evaluation
Severe stress	2.8 (1)	5.6 (2)	2.8 (1)	13.8 (5)	13.9 (5)	11.1 (4)
Medium stress	27.8 (10)	19.4 (7)	27.8 (10)	55.6 (20)	55.6 (20)	36.1 (13)
Little stress	69.4 (25)	75.0 (27)	69.4 (25)	30.6 (11)	30.6 (11)	52.8 (11)

### TAPS: psychometric properties

We determined the floor and ceiling effects and indicated none of the investigated females with AIS scored the TAPS minimum total score during the 1st, 2nd and 3rd assessments. Only one study participant (2.7 %) scored the maximum (5 points) during the 3rd assessment. The Cronbach’s alpha values for the general result of TAPS equaled 0.84, 0.78 and 0.50 in the 1st, 2nd and 3rd evaluation, respectively. The analysis of discriminant validity, as assessed by calculating the correlation between the total TAPS score and the Cobb angle is summarized in Table 7.

### Responsiveness of TAPS, SRS-22 and BSSQ

To quantify the responsiveness of the TAPS, SRS-22 and BSSQ general results, effect sizes were calculated for each measure to assess the magnitude of the treatment effect. The effect size concerning the 2nd and 1st evaluation in regards to TAPS, SRS-22, BSSQ-Brace and BSSQ-Deformity total scores was small for TAPS only and equaled 0.343. The remaining values for SRS-22 and BSSQ were below 0.3. The effect size for the 3rd and 1st evaluation was medium for TAPS, and small for SRS-22 and BSSQ-Brace and equaled 0.527, 0.328 and 0.300, respectively. The effect size for BSSQ-Deformity was below the value of 0.3. The effect size concerning the 3rd and 2nd evaluation was small for SRS-22 and BSSQ-Brace (0.440 and 0.313, respectively); the values of the effect size for TAPS and BSSQ-Deformity were below 0.3.

### The minimum clinically important difference (MCID) of the SRS-22

The SEM value equaled 0.141 for the SRS-22 total score, 0.283 for function/activity, 0.203 for pain, 0.311 for self-image, 0.236 for mental health and 0.262 for satisfaction with treatment domains. The condition of patients with a change score above these values was considered either minimally improved or minimally worse in the respective SRS-22 total and domain scores.

### Differences between the 1st, 2nd and 3rd patient evaluation

The multiple comparisons, by means of the Friedman two-way ANOVA, indicated the significant differences concerned TAPS Figure 2 (*p* = 0.012), Figure 3 (*p* = 0.013) and the total score (*p* = 0.013). Furthermore, differences concerned BSSQ-Brace items 5 and 6 (for details, see Table 4). Wilcoxon signed-rank tests were then used to compare the TAPS, SRS-22 and BSSQ results across the three assessment times. A Bonferroni adjustment for three comparisons was made and *p* = 0.017 was set as the border level of statistical significance. In regards to TAPS, results differed between the 1st and the 2nd assessment in Figure 2 only (*p* = 0.013). The difference between the 1st and the 3rd evaluation concerned Figure 3 and the total score (*p* = 0.011 and *p* = 0.005, respectively) (Table 5). Study participants’ SRS-22 and BSSQ results did not differ significantly between the 1st and the 2nd, between the 2nd and the 3rd and between the 1st and the 3rd evaluation (for details, see Tables 5, 6).

**Table 4:** Results of multiple comparisons between the 1st, the 2nd and the 3rd completion of Trunk Appearance Perception Scale, Scoliosis Research Society-22 and Bad Sobberheim Stress Questionnaires

Trunk Appearance Perception Scale				Scoliosis Research Society-22					
Figure 1	Figure 2	Figure 3	Total score	Function/activity	Pain	Self-image	Mental health	Satisfaction from treatment	Total score
*p* = 0.106	*p* = 0.012	*p* = 0.013	*p* = 0.013	*p* = 0.612	*p* = 0.209	*p* = 0.127	*p* = 00.419	*p* = 0.924	*p* = 0.087

Bad Sobberheim Stress Questionnaire-Deformity								
Item 1	Item 2	Item 3	Item 4	Item 5	Item 6	Item 7	Item 8	Total score
*p* = 0.497	*p* = 0.492	*p* = 0.957	*p* = 0.988	*p* = 0.721	*p* = 0.304	*p* = 0.815	*p* = 0.513	*p* = 0.621

Bad Sobberheim Stress Questionnaire-Brace								
Item 1	Item 2	Item 3	Item 4	Item 5	Item 6	Item 7	Item 8	Total score
*p* = 0.350	*p* = 0.104	*p* = 0.079	*p* = 0.603	*p* = 0.038	*p* = 0.008	*p* = 0.637	*p* = 0.404	*p* = 0.153

**Table 5:** Comparisons between the 1st, the 2nd and the 3rd completion of Trunk Appearance Perception Scale and Scoliosis Research Society-22

Trunk Appearance Perception Scale				Scoliosis Research Society-22					
Figure 1	Figure 2	Figure 3	Total score	Function/activity	Pain	Self-image	Mental health	Satisfaction from treatment	Total score
*Comparison: 1st evaluation–2nd evaluation*									
*p* = 0.118	*p* = 0.013*	*p* = 0.435	*p* = 0.124	*p* = 0.400	*p* = 0.170	*p* = 0.459	*p* = 0.773	*p* = 0.959	*p* = 0.768
*Comparison: 2nd evaluation–3rd evaluation*									
*p* = 0.077	*p* = 0.021	*p* = 0.011*	*p* = 0.005*	*p* = 0.848	*p* = 0.019	*p* = 0.094	*p* = 0.254	*p* = 0.590	*p* = 0.029
*Comparison: 1st evaluation–3rd evaluation*									
*p* = 0.767	*p* = 0.374	*p* = 0.049	*p* = 0.168	*p* = 0.501	*p* = 0.584	*p* = 0.114	*p* = 0.046	*p* = 0.627	*p* = 0.065

* *p* < 0.017 (after adjustment to Bonferroni correction)

**Table 6:** Results of comparisons according to individual items and total scores between the 1st, 2nd and 3rd Bad Sobberheim Stress Questionnaire-Deformity and Bad Sobberheim Stress Questionnaire-Brace results

Bad Sobberheim Stress Questionnaire	Item 1	Item 2	Item 3	Item 4	Item 5	Item 6	Item 7	Item 8	Total score
*Comparison: 1st evaluation–2nd evaluation*									
Bad Sobberheim Stress Questionnaire-Deformity	*p* = 0.496	*p* = 0.925	*p* = 0.955	*p* = 0.760	*p* = 0.755	*p* = 0.209	*p* = 1.000	*p* = 0.638	*p* = 0.649
Bad Sobberheim Stress Questionnaire-Brace	*p* = 0.670	*p* = 0.467	*p* = 0.972	*p* = 0.823	*p* = 0.527	*p* = 0.065	*p* = 0.396	*p* = 0.367	*p* = 0.666
*Comparison: 2nd evaluation–3rd evaluation*									
Bad Sobberheim Stress Questionnaire-Deformity	*p* = 0.836	*p* = 0.397	*p* = 0.868	*p* = 0.717	*p* = 0.950	*p* = 0.977	*p* = 0.333	*p* = 0.272	*p* = 0.638
Bad Sobberheim Stress Questionnaire-Brace	*p* = 0.463	*p* = 0.069	*p* = 0.083	*p* = 0.545	*p* = 0.046	*p* = 0.286	*p* = 0.542	*p* = 0.263	*p* = 0.063
*Comparison: 1st evaluation–3rd evaluation*									
Bad Sobberheim Stress Questionnaire-Deformity	*p* = 0.287	*p* = 0.379	*p* = 0.826	*p* = 0.961	*p* = 1.000	*p* = 0.300	*p* = 0.351	*p* = 0.445	*p* = 0.427
Bad Sobberheim Stress Questionnaire-Brace	*p* = 0.756	*p* = 0.050	*p* = 0.136	*p* = 0.638	*p* = 0.072	*p* = 0.026	*p* = 0.754	*p* = 0.784	*p* = 0.111

### Associations between characteristics of females with AIS and questionnaire results in the course of brace treatment

Having analyzed the correlation between characteristics of females with AIS and TAPS results in the 1st evaluation, we indicated a significant but moderate correlation between the Cobb angle and the total score and Figures 1 and 2 (rs = −0.46, rs = −0.46 and rs = −0.44) (Table 7). In the 1st SRS-22 assessment, thoracic apical translation was related to the function/activity, mental health domains and the total score (rs = −0.40, rs = −0.44 and rs = −0.49, respectively). Age of female study participants was related to mental health and the total score (rs = −0.41, rs = −0.41, respectively). In the 1st evaluation by means of BSSQ, the only significant association emerged with apical translation and both BSSQ-Deformity and BSSQ-Brace total scores (rs = −0.42, *p* = 0.011; rs = −0.37, *p* = 0.026).

**Table 7:** Correlation between Trunk Appearance Perception Scale, Scoliosis Research Society-22 and Bad Sobberheim Stress Questionnaires results and patients characteristics during the 1st, 2nd and the 3rd assessment

Patient characteristics	TAPS				SRS-22						BSSQ-Deformity	BSSQ-Brace
	Figure 1	Figure 2	Figure 3	Total score	Function/activity	Pain	Self-image	Mental health	Satisfaction with treatment	Total score		
Body mass index	rs = 0.01	rs = −0.03	rs = −0.16	rs = −0.01	rs = −0.20	rs = 0.07	rs = 0.03	rs = −0.21	rs = 0.23	rs = −0.05	rs = 0.09	rs = −0.13
	rs = 0.18	rs = −0.16	rs = −0.02	rs = −0.07	rs = −0.06	rs = −0.24	rs = 0.04	rs = −0.05	rs = −0.22	rs = −0.17	rs = −0.19	rs = −0.25
	rs = 0.20	rs = −0.07	rs = −0.12	rs = −0.03	rs = 0.19	rs = −0.29	rs = 0.01	rs = −0.21	rs = −0.29	rs = −0.11	rs = −0.14	rs = −0.16
Age	rs = −0.01	rs = −0.09	rs = 0.05	rs = 0.01	rs = −0.11	rs = −0.33	rs = −0.18	rs = −0.41*	rs = −0.10	rs = −0.41*	rs = −0.13	rs = −0.23
	rs = 0.15	rs = 0.25	rs = −0.11	rs = 0.12	rs = −0.48*	rs = −0.24	rs = 0.16	rs = −0.18	rs = 0.10	rs = −0.22	rs = −0.18	rs = −0.23
	rs = 0.15	rs = 0.10	rs = −0.34*	rs = −0.12	rs = −0.23	rs = −0.35*	rs = 0.07	rs = −0.49*	rs = −0.12	rs = −0.30	rs = −0.02	rs = −0.04
Cobb angle	rs = −0.46*	rs = −0.46*	rs = −0.25	rs = −0.44*	rs = −0.16	rs = 0.08	rs = 0.01	rs = −0.26	rs = −0.16	rs = −0.21	rs = −0.10	rs = −0.10
	rs = −0.03	rs = −0.07	rs = 0.12	rs = 0.10	rs = −0.31	rs = −0.09	rs = 0.01	rs = −0.09	rs = −0.09	rs = −0.19	rs = −0.20	rs = −0.33*
Angle of trunk rotation as measured with Perdriolli’s inclinometer	rs = −0.14	rs = −0.16	rs = −0.01	rs = −0.14	rs = −0.13	rs = 0.14	rs = 0.08	rs = −0.05	rs = −0.17	rs = −0.06	rs = −0.29	rs = 0.01
	rs = 0.01	rs = −0.43*	rs = −0.12	rs = −0.28	rs = 0.15	rs = 0.40*	rs = 0.02	rs = 0.24	rs = 0.05	rs = 0.30	rs = −0.28	rs = −0.09
	rs = −0.15	rs = 0.40*	rs = 0.03	rs = −0.17	rs = −0.03	rs = 0.20	rs = 0.05	rs = 0.08	rs = −0.26	rs = 0.11	rs = −0.15	rs = −0.21
Apical translation of the central sacral vertical line (CSVL) according to the Harms Study Group (cm)	rs = −0.27	rs = −0.32	rs = −0.12	rs = −0.26	rs = −0.40*	rs = −0.19	rs = −0.32	rs = −0.44*	rs = 0.06	rs = −0.49*	rs = −0.42*	rs = −0.37*
	rs = −0.01	rs = −0.02	rs = 0.07	rs = 0.09	rs = −0.45*	rs = −0.21	rs = 0.03	rs = −0.35*	rs = −0.03	rs = −0.31	rs = −0.26	rs = −0.29
Brace (hours/day)	rs = −0.07	rs = 0.07	rs = −0.07	rs = −0.01	rs = −0.23	rs = 0.09	rs = −0.01	rs = −0.02	rs = 0.31	rs = 0.01	rs = −0.03	rs = −0.04
	rs = −0.08	rs = −0.21	rs = 0.02	rs = −0.09	rs = −0.13	rs = 0.04	rs = −0.01	rs = 0.11	rs = 0.01	rs = 0.03	rs = −0.06	rs = −0.01
	rs = 0.28	rs = 0.16	rs = 0.09	rs = 0.23	rs = −0.09	rs = −0.07	rs = −0.04	rs = 0.06	rs = 0.31	rs = 0.01	rs = −0.09	rs = 0.12
Brace (in months)	rs = 0.22	rs = −0.09	rs = 0.09	rs = 0.06	rs = −0.12	rs = 0.01	rs = 0.11	rs = 0.07	rs = −0.09	rs = 0.02	rs = −0.05	rs = −0.09
	rs = 0.26	rs = −0.03	rs = 0.04	rs = 0.01	rs = −0.02	rs = −0.09	rs = −0.05	rs = 0.09	rs = −0.10	rs = −0.01	rs = 0.06	rs = −0.24
	rs = 0.18	rs = −0.31	rs = −0.20	rs = −0.17	rs = −0.03	rs = −0.01	rs = 0.08	rs = −0.05	rs = −0.08	rs = −0.06	rs = −0.15	rs = −0.15

The top row contains results from the 1st assessment, the middle row from the 2nd assessment and the bottom row from the 3rd assessment with the exception of results for Cobb angle and apical translation which are given for the 1st and the 3rd assessment

* *p* < 0.05

Having analyzed the correlation between characteristics of females with AIS and TAPS results in the 2nd evaluation, we indicated a moderate correlation between Figure 2 and the angle of trunk rotation (rs = −0.43) only. In the 2nd SRS-22 evaluation, the age of the study participants was significantly associated with the function/activity domain (rs = −0.48), whereas angle of trunk rotation was related with pain subscale (rs = −0.40).

In the last evaluation, a correlation between TAPS Figure 2 and angle of trunk rotation (rs = −0.40) and between age on bracing and Figure 3 (rs = −0.34) was indicated. Furthermore, age was associated with the pain and mental health SRS-22 subscales (rs = −0.35, rs = −0.49, respectively), whereas thoracic apical translation correlated to function/activity and mental health (rs = −0.45 and rs = −0.35, respectively). Moreover, we supported a significant correlation between the Cobb angle and BSSQ-Brace results (rs = 0.33, *p* = 0.047) (for details, see Table 7).

### Longitudinal analysis of associations between TAPS, SRS-22 Questionnaire and BSSQ

We analyzed the relations between questionnaire results longitudinally and indicated a significant, moderate association between TAPS Figure 3 and the SRS-22 function/activity domain in the 3rd evaluation (rs = 0.34) (see Table 8).

**Table 8:** Associations between Trunk Appearance Perception Scale, Scoliosis Research Society-22 and Bad Sobberheim Stress Questionnaires results in the course of brace treatment

Trunk Appearance Perception Scale	Scoliosis Research Society-22						Bad Sobberheim Stress Questionnaires	
	Function/activity	Pain	Self-image	Mental health	Satisfaction with treatment	Total score	Bad Sobberheim Stress Questionnaire-Deformity	Bad Sobberheim Stress Questionnaire-Deformity
*1st assessment*								
Figure 1	rs = −0.09	rs = −0.07	rs = 0.16	rs = −0.01	rs = 0.07	rs = 0.01	rs = −0.08	rs = −0.18
Figure 2	rs = 0.01	rs = −0.20	rs = 0.05	rs = 0.07	rs = 0.06	rs = 0.02	rs = 0.09	rs = 0.04
Figure 3	rs = 0.05	rs = −0.06	rs = 0.15	rs = 0.17	rs = 0.15	rs = 0.13	rs = 0.12	rs = −0.01
Total score	rs = −0.06	rs = −0.16	rs = 0.09	rs = 0.01	rs = 0.20	rs = −0.01	rs = 0.04	rs = −0.08
*2nd assessment*								
Figure 1	rs = −0.07	rs = −0.08	rs = 0.15	rs = −0.03	rs = −0.24	rs = 0.04	rs = 0.02	rs = −0.03
Figure 2	rs = −0.06	rs = −0.03	rs = 0.07	rs = −0.26	rs = −0.10	rs = −0.12	rs = 0.08	rs = 0.08
Figure 3	rs = 0.06	rs = 0.29	rs = 0.24	rs = 0.07	rs = −0.14	rs = 0.27	rs = 0.30	rs = 0.27
Total score	rs = 0.02	rs = 0.19	rs = 0.21	rs = −0.04	rs = −0.15	rs = 0.16	rs = 0.20	rs = 0.26
*3rd assessment*								
Figure 1	rs = 0.07	rs = 0.19	rs = 0.09	rs = −0.17	rs = 0.10	rs = −0.09	rs = −0.01	rs = −0.16
Figure 2	rs = 0.22	rs = −0.07	rs = 0.09	rs = 0.12	rs = 0.14	rs = 0.09	rs = 0.12	rs = −0.03
Figure 3	rs = 0.34*	rs = 0.16	rs = 0.14	rs = 0.27	rs = 0.23	rs = 0.30	rs = 0.17	rs = 0.01
Total score	rs = 0.30	rs = 0.08	rs = 0.22	rs = 0.25	rs = 0.31	rs = 0.27	rs = 0.24	rs = −0.05

* *p* < 0.05

SRS-22 correlated to BSSQ-Deformity total score in all the evaluations of females with AIS participating in the study (rs = 0.60, *p* < 0,001; rs = 0.35, *p* = 0,037; rs = 0.69, *p* < 0,001, respectively), whereas SRS-22 was associated with BSSQ-Brace in the 1st (rs = 0.62, *p* < 0,001) and 2nd assessment (rs = 0.38, *p* = 0.21). Furthermore, the BSSQ-Brace total score strongly correlated to the BSSQ-Deformity results during the 1st evaluation (rs = 0.60, *p* < 0.001), whereas the association between BSSQ total scores during the 3rd assessment was moderate (rs = 0.49, *p* = 0.002) (for detailed analysis of associations between SRS-22 subscales and BSSQ scores, see Table 9).

**Table 9:** Associations between Scoliosis Research Society-22 and Bad Sobberheim Stress Questionnaires results in the course of brace treatment

Scoliosis Research Society-22	Function/activity	Pain	Self-image	Mental health	Satisfaction with treatment	Total score	Bad Sobberheim Stress Questionnaire-Deformity
*1st assessment*							
Bad Sobberheim Stress Questionnaire-Deformity	rs = 0.39*	rs = 0.17	rs = 0.45*	rs = 0.54*	rs = 0.25	rs = 0.60*	–
Bad Sobberheim Stress Questionnaire-Brace	rs = 0.44*	rs = 0.26	rs = 0.48*	rs = 0.48*	rs = 0.22	rs = 0.62*	rs = 0.60*
*2nd assessment*							
Bad Sobberheim Stress Questionnaire-Deformity	rs = 0.18	rs = −0.03	rs = 0.46*	rs = 0.44*	rs = 0.12	rs = 0.35*	–
Bad Sobberheim Stress Questionnaire-Brace	rs = 0.62*	rs = 0.13	rs = 0.19	rs = 0.26	rs = 0.01	rs = 0.38*	rs = 0.18
*3rd assessment*							
Bad Sobberheim Stress Questionnaire-Deformity	rs = 0.44*	rs = 0.40*	rs = 0.62*	rs = 0.62*	rs = 0.16	rs = 0.69*	–
Bad Sobberheim Stress Questionnaire-Brace	rs = 0.37*	rs = 0.20*	rs = 0.07	rs = 0.29	rs = 0.27	rs = 0.32	rs = 0.49*

* *p* < 0.05

### Regression analyses

The regression analyses were performed after subjects were grouped by duration of brace wearing in months as follows: 0–12 months, 13–24 months and >24 months.

The logistic regression model obtained as a result of the calculations revealed that none of the investigated variables had a statistically significant influence on the probability of achieving a “good” total TAPS score as well as a “good” result in the SRS-22 in all assessment stages.

In regards to BSSQ-Deformity, it is interesting to note that the regression analysis revealed that only the BSSQ-Brace had a statistically significant (*p* = 0.033) influence on the probability of females reporting a low level of deformity-related stress during the final evaluation. An increase in 1 point in the BSSQ-Brace total score increased the probability of achieving a low stress level by 19 %. Furthermore, in the last assessment, only BSSQ-Deformity total score significantly (*p* = 0.015) influenced the probability of females reporting a low level of brace-related stress, as measured by the BSSQ-Brace. An increase in 1 point in the BSSQ-Deformity total score increased the probability of achieving a low stress level by 44 %. None of the remaining investigated variables had a statistical influence on the probability of achieving a “good” result both for the BSSQ-Deformity and BSSQ-Brace, during all assessment times.

## Discussion

The purpose of this longitudinal study was to assess changes over time in emotional stress, self- and body image, mental health and perception of body functioning, including pain, in adolescent females with scoliosis treated conservatively.

To assess the outcome of conservative therapeutic intervention in adolescent females, it seems essential to measure the changes in girls’ own perceptions of scoliosis- and brace-related emotional stress, spinal appearance and mental health, according to the duration of brace wearing in months and progression or stabilization of the disease. Our assumptions are that the monitoring of the functioning of females with AIS should be routinely implemented during brace treatment, since the longitudinal exploration of the perception of disease and psychopathological changes in the course of brace treatment would shed new light on factors determining their quality of life and, therefore, constitute useful practical implications for individual support programs. Furthermore, it is considered necessary to investigate the emotional burden investigated females with AIS experience due to spinal disfigurement and orthosis wearing carefully over the course of orthotic treatment in order to provide psychological support and to increase compliance with conservative treatment, essential for a successful outcome.

The prospective, threefold assessment of body image was based on detailed, self-evaluation of views of the trunk from behind and in the axial plane as well as a frontal view, which probably corresponds to the most realistic perception of one’s body [20]. We believe such analysis in terms of assessing changes in quality-of-life data over time, age on bracing, as well as clinical and radiological data related to progression or stabilization of the disease, is of particular importance in the prevention of body-image disturbances.

Only female patients were included in the studied group. As mentioned earlier, adolescent females tend to be more commonly affected with scoliosis, than males [14]. Furthermore, we decided not to include one male with AIS in the analysis because of the high probability that the sample group size of males with AIS would be inadequate. The same selection criterion of choosing female subjects only for a study was applied by Noonan et al. [9]. The interpretation of the results from this study in the light of findings from earlier research requires caution since previous studies included both male and female participants, whereas the present study focuses only on females with AIS. Gratz et al. [8] indicated that it is more common for young females with scoliosis to give up activities they enjoyed doing or socializing with peers due to negative body image than young males. Sapountzi-Krepia et al. [28] found that females had a more disturbed body image than males, and compared with the healthy population had lower levels of happiness and life satisfaction. In addition, Aulisa et al. [29] found that in regard to areas, such as mood, social relationships, stress, school activity, social functioning, perception of one’s own health state, emotional functioning, self-esteem, a sense of esthetics, vitality and level of pain, males had higher positive scores than females. Based on the results derived from previous research, we hypothesize the differences between males and females in regards to TAPS, SRS-22 and BSSQ scores would occur in favor of male patients. In addition, discrepancies concerning the first and last completion of TAPS would not be expected to be as high as for females with scoliosis. However, this issue warrants further research.

Kenneth et al. [30] in a study on the effects of brace treatment by means of SRS-22 results, indicated there was a positive correlation of function/activity and pain with bracing time, and a negative correlation of self-image, mental health, satisfaction and total score with monthly duration of brace wearing, suggesting that function/activity improved with the time the brace is worn in months, but image/appearance decreased with time spent in the brace in months which is in contrast to our analysis in terms of the significance of the differences between the 1st, 2nd and last evaluation by means of SRS-22. Interestingly, accounting for the TAPS results, it was revealed the disparities between the 1st and 3rd assessments were, as expected, the strongest, especially in regards to Figures 2 (the patient bending forward seen from the front—Adam’s test), Figure 3 (frontal view) and the TAPS total score, suggesting positive changes over time concerning girls’ body image. Taking into account the dynamics of stress level and the particular coping efforts, patients applied in the course of a 12-month observation treatment period—we did not record any significant deterioration or improvement in the functioning of females with AIS in terms of perceived bracing- and deformity-related stress levels, this does not comply with reports supporting the positive role of the length of conservative treatment in months for adjustment to brace treatment and spinal disfigurement [10, 16]. Furthermore, the regression analysis after grouping the subjects by duration of brace wearing in months did not reveal the influence of monthly duration of brace wearing on the probability of reporting a low deformity- and orthosis wearing-related stress by AIS females. However, it must be emphasized this analysis revealed that the level of brace-related stress significantly influenced deformity-related stress intensity, and a reverse dependency was also confirmed, both after a 12-month treatment observation period. These results directed attention to the mutual influence of experienced stress due to body disfigurement as well as the necessity for conservative treatment in a population of adolescent female patients with AIS.

Kenneth et al. suggested that the reduction in quality of life increasing with monthly duration of brace wearing may be explained by the fact that as the study participants grew older they became more self-aware and had higher expectations of their social life, hence the decreasing scores in self-image, mental health and satisfaction with monthly duration of brace wearing, while as they got used to wearing the brace function and pain scores improved [30]. However, it was shown in the present study that younger girls constituted the risk group of psychological impairment, since they scored lower in regards to body image, mental health, pain, function/activity and in general assessment of their functioning. Furthermore, the most self-criticism of investigated females regarded self-image and the back view of the trunk. The mentioned results indicated the sphere of psychosocial functioning that was of special importance for psychosocial disturbances which, therefore, should be taken into account in planning individual and group scoliosis support programs.

In a study by Rigo et al., the authors similarly, as in the current research, analyzed the interrelationships between SRS-22 and TAPS scores in AIS females and indicated a significant positive correlation between females’ TAPS, and self-image and pain domains in the SRS-22 results [31]. Interestingly, the results of our study showed only one significant association between nonverbal assessment of body shape by means of images contained in the TAPS and particular domains of SRS-22, indicating minimal or no relation between adolescents’ ability to perform everyday activities, pain level or mood disturbances and tendency to positively assess body shape and physical attractiveness. However, strong associations between bracing- and deformity-related stress level and SRS-22 results have been confirmed, especially in regards to self-image, activity and mental health.

It is very common to encounter discrepancies between the radiological evaluation of deformity in terms of Cobb angle and females with AIS own evaluations of esthetic deformity. Interestingly, the presented study supported the associations between the size of the deformity in terms of radiological data, such as apical translation and Cobb angle, and the stress level in general. However, these associations regarded only the first and last assessment. The presented results correspond to the Weiss et al. study, which assessed the psychological stress scoliosis patients develop as a consequence of their deformity in a sample with an average Cobb angle of 35.8 degrees and indicated that the level of stress correlated with the Cobb angle (*r* = −0.54; *p* < 0.001) [6]. Moreover, our study indicated associations between the radiological evaluations of spinal deformity and females’ subjective evaluations of body appearance, showing the influence of the angle of trunk rotation and Cobb angle on TAPS results. Mental health and function/activity as measured by the SRS-22 were related to thoracic apical translation.

Particularly important as far as practical implications are concerned, since the severity of spinal disfigurement as well as negative self-image constitute a potential risk leading to emotional distress, it ought to be one of the factors taken into account when considering psychological screening and in providing appropriate support for AIS females. We postulate more attention should be paid to the physical and psychosocial impact of brace treatment among female adolescents, especially for younger girls and those with severe spinal deformity confirmed in clinical and radiological evaluation at the beginning of conservative treatment.

The minimum clinically important difference (MCID), recognized as an improvement threshold clinically relevant to the individual patient, is increasingly used to evaluate treatment effectiveness [32]. Referring to the clinical relevance of this prospective research concerning conservatively treated patients, the minimum clinically important difference for the SRS-22 was calculated to help clinicians interpret whether the changes in SRS-22 scores over time are both statistically significant and clinically meaningful for individual, brace-treated adolescent females. Carreon et al. determined the MCID of the SRS-22 self-image, activity and pain domains, although this was for a group of males and females with AIS undergoing surgical correction. The MCID for the pain domain was 0.20 and 0.98 for self-image domain. A minimal though significant change in the activity subscale was also observed (0.08) [32]. Interestingly, our study concerning changes in AIS females’ functioning in the course of brace treatment regimen revealed the highest MCID value emerged in the self-image subscale, which is congruent with the findings by Carreon et al. [32]. The aforementioned results point out that the highest expectations and most important change, considered as clinically significant for an individual patient in the course of AIS treatment, applies to body appearance. We believe the MCID calculations are of particular importance, since our study concerning analysis of changes in AIS females’ functioning, applying to the whole patient group, did not reveal statistically significant differences referring to SRS-22 total score and particular subscales, leading to conclusion that self-perceived health status does not deteriorate or improve in the course of orthosis treatment. However, this may not reflect clinically significant changes within individual patient scores.

Some limitations of the present study should be pointed out. It is necessary to underline that only female patients were investigated in the study. Considering external validity, the current study’s sample characteristics limit the generalizability of the presented findings concerning changes over time in the perception of patients’ psychosocial functioning to adolescent females subjected for an underarm brace treatment in a short, 12-month follow-up only. To expand the generalizability of the current study, we postulate future studies would examine males and females with AIS in a longer follow-up, as well as scoliosis patients of less than 20 and over 45 degrees, subjected to surgical treatment. Furthermore, as TAPS showed the highest responsiveness compared with the SRS-22 and BSSQ, which was supported in the current study and indicates this instrument is suitable for the evaluation of changes in patients’ functioning over time and the magnitude of a treatment effect, future research would benefit from including a nonverbal assessment of spinal appearance into a longitudinal analysis of changes in emotional stress, spinal appearance and mental health in the aforementioned study populations.

## Conclusions

The study indicated that the assessment of girls with AIS concerning body function and mental health did not deteriorate in the course of orthosis treatment. Furthermore, they showed improvement in perceptions particularly in regards to trunk shape. We pointed out that the negative perceptions of mental health, self-image and low level of activity held by females with AIS coexisted with severe emotional distress. Moreover, factors that improved functioning or subjective physical appearance ratings in particular, such as level of activity, were indicated.
